# Predictors of smoking cessation treatment attrition in individuals with substance use disorders

**DOI:** 10.1192/j.eurpsy.2021.2175

**Published:** 2021-08-13

**Authors:** G. Aonso-Diego, Á. García-Pérez, S. Weidberg, R. Secades-Villa

**Affiliations:** Department Of Psychology, University of Oviedo, Oviedo, Spain

**Keywords:** Substance Use Disorder, attrition, dropouts, smoking cessation

## Abstract

**Introduction:**

Attrition rates in smoking cessation treatments are high, particularly in persons with substance use disorders. It is estimated that about 55%% disengage prematurely at treatment, meaning that a large portion will not benefit from smoking abstinence. So far, no previous studies have examined predictors of dropouts in a smoking cessation treatment with persons with SUD.

**Objectives:**

The study was two-fold: 1) to analyze the percentage of early-, late-dropouts and completers, and 2) to examine sociodemographic, psychological, and substance-related predictors of dropouts.

**Methods:**

A total of 86 participants (69.8% males; M_age_=43.84, SD=9.917) were randomly assigned to two psychological smoking cessation treatment: cognitive-behavioral treatment (CBT) (n=51) or CBT + contingency management (CM) (n=35). Interventions were delivered during eight consecutive weeks

**Results:**

Of the 86 participants who completed the baseline assessment, 21 did not start treatment, 17 dropped out of treatment during treatment, and the remaining 48 completed the treatment. Predictors of early-dropout were younger age (B=-.234; p=.024; OR=.792) and lower number of days in SUD treatment (B= -.005; p=.026; OR=.995). Patients’ primary substance of use was associated with reduced early-dropouts; compared to cocaine users, alcohol (B=-1.827; p=.043; OR=.161) and opioids (B=-3.408; p=.018; OR=.033) related to improved attrition. Late dropout was directly related to higher number of tobacco use cessation attempts (B=.407; p=.039; OR=1.502).

**Conclusions:**

Incorporating strategies to improve attendance and completion rates in SUD populations should be a priority. Mobile reminders, offering online therapies, or CM to reinforce attendance to therapy may be considered.

**Disclosure:**

No significant relationships.

